# Th-1, Th-2 Cytokines Profile among *Madurella mycetomatis* Eumycetoma Patients

**DOI:** 10.1371/journal.pntd.0004862

**Published:** 2016-07-19

**Authors:** Amre Nasr, Amir Abushouk, Anhar Hamza, Emmanuel Siddig, Ahmed H. Fahal

**Affiliations:** 1 Department of Basic Medical Sciences, College of Medicine, King Saud Bin Abdulaziz University for Health Sciences, Riyadh, Kingdom of Saudi Arabia; 2 King Abdullah International Medical Research Centre, National Guard Health Affairs, Kingdom of Saudi Arabia; 3 Department of Microbiology, College of Sciences and Technology, Al-Neelain University, Khartoum, Sudan; 4 Department of Medical Protocol, King Abdulaziz Medical City – Riyadh, Ministry of National Guard – Health Affairs, Riyadh, Kingdom of Saudi Arabia; 5 Mycetoma Research Centre, University of Khartoum, Khartoum, Sudan; University of California San Diego School of Medicine, UNITED STATES

## Abstract

Eumycetoma is a progressive and destructive chronic granulomatous subcutaneous inflammatory disease caused by certain fungi, the most common being *Madurella mycetomatis*. The host defence mechanisms against fungi usually range from an early non-specific immune response to activation and induction of specific adaptive immune responses by the production of Th-1 and Th-2 cytokines. The aim of this study is to determine the levels of Th-1 and Th-2 cytokines in patients infected with *Madurella mycetomatis*, and the association between their levels and disease prognosis. This is a descriptive cross-sectional study conducted at the Mycetoma Research Centre, University of Khartoum, Sudan, where 70 patients with confirmed *M*. *mycetomatis* eumycetoma were enrolled; 35 with, and 35 without surgical excision. 70 healthy individuals from mycetoma endemic areas were selected as controls. The levels of serum cytokines were determined by cytometric bead array technique. Significantly higher levels of the Th-1 cytokines (IFN-γ, TNF-α, IL-1β and IL-2) were recorded in patients treated with surgical excision, compared to those treated without surgical excision. In contrast, the Th-2 cytokines (IL-4, IL-5, IL-6 and IL-10) were significantly lower in patients treated with surgical excision compared to those treated without surgical excision. In conclusion, the results of this study suggest that cell-mediated immunity can have a role to play in the pathogenesis of eumycetoma.

## Author Summary

*Madurella mycetomatis* is the most common causative agent for eumycetoma, which is a progressive and destructive subcutaneous inflammatory disease. It is a neglected tropical disease affecting the population in poor and remote endemic tropical and subtropical areas. Currently, the susceptibility and resistance to mycetoma are not well defined, and many factors can be incriminated, including immunological, genetic, or environmental ones. The current descriptive cross-sectional study was conducted to determine the Th-1 and Th-2 cytokine levels among 70 patients with *Madurella mycetomatis* eumycetoma and 70 healthy controls. It aimed to find out the association between the disease prognosis and the level of these cytokines. Significantly higher levels of the Th-1 cytokines (IFN-γ, TNF-α, IL-1β and IL-2) were found in patients treated with surgical excision compared to those treated without surgical intervention. However, the Th-2 cytokines (IL-4, IL-5, IL-6 and IL-10) were significantly lower in patients treated with surgical excision compared to those treated without surgical excision. These findings suggested that, cell-mediated immunity has a prime role in the pathogenesis of eumycetoma.

## Introduction

Mycetoma is a chronic subcutaneous infection caused by certain bacteria (actinomycetoma) or fungi (eumycetoma) [1]. It is characterised by a slow progressive infection and a granulomatous inflammatory response that can result in severe soft tissue and muscle damage along with destruction of the underlying bone [1, 2]. Mycetoma is endemic in tropical and subtropical regions; however, it has been reported globally. Eumycetoma in Sudan, is predominately caused by the fungus *Madurella mycetomatis* [2]. The disease is characterised by extensive subcutaneous masses, usually with multiple draining sinuses and fungal grains [1]. Mycetoma disease has significant negative medical health and socio-economic impacts on patients and communities, affects individuals of all ages, but is more frequently seen in adults who work outdoors.

The host defence mechanisms against fungi usually range from germline encoded immunity which present early in the evolution of microorganisms, to highly specialised and specific adaptive mechanisms that are induced by infection and disease. The innate response to fungi serves two main purposes; a direct antifungal effector activity and activation or induction of specific adaptive immune responses. In general, the direct antifungal effector activity mediates non-specific elimination of pathogens through either a phagocytic process with intracellular killing of internalised pathogens or through the secretion of microbiocidal compounds against undigested fungal molecules. The activation and induction of the specific adaptive immune responses is accomplished by the production of pro-inflammatory mediators, including chemokines and cytokines, providing co-stimulatory signals to naive T cells, as well as antigen uptake and presentation to CD4^+^ and CD8^+^ T cells [3, 4].

Many individuals in mycetoma endemic areas are exposed to the causative aetiological agents, but only few develop the disease. This may suggest variable responses of the host immune system towards the invading agent. In this respect, the role of the innate immunity in host resistance to mycetoma infection has been studied *in vitro* and in animal models, but few studies have been performed in humans.

T cell–mediated immune response to eumycetoma fungi in humans was studied by Mahgoub and associates who suggest that patients with eumycetoma have a weak cell-mediated response as determined by skin reaction to dinitrochlorobenzene [5]. Decreased lymphocyte proliferative response to phytohemagglutinin in those patients was also reported. However, no evidence was provided to confirm whether this is a primary immune deficiency or a secondary response to a severe infection. In addition, the same study showed high levels of IgA and IgM and low levels of IgG antibodies in mycetoma patients [5].

In actinomycetoma, Gonzalez-Ochoa and Baranda [6] found that patients with severe lesions and extensive tissue destruction displayed a weak skin reaction to some pathogenic bacteria polysaccharides such as, *Nocardia brasiliensis* [6]. However, it was not clear whether this represented a T-helper-1 (Th-1) or T-helper-2 (Th-2) response. To date there has been limited data on the immune response to mycetoma infection and how patients can modulate their response against *M*. *mycetomatis*.

With this background, the present study aims to determine the Th-1 and Th-2 cytokines response of patients infected with *M*. *mycetomatis* and to find out the association between the measured Th-1 and Th-2 cytokine levels and the disease prognosis and outcome.

## Materials and Methods

### Study population

This descriptive cross-sectional hospital based study was conducted at the Mycetoma Research Centre, University of Khartoum, Khartoum, Sudan. In this study 140 individuals were enrolled; 49 (35%) were females and 91 (65%) were males (Table 1), with an overall median age of 25 years (range 12–70 years). 70 patients with confirmed mycetoma infection due to *Madurella mycetomatis* were recruited. The study population was divided into three groups; **group I:** healthy controls (n = 70; median age 25 years (range 12 to 70 years)), matched for sex, age and locality with the patients group. **Group II:** mycetoma patients without surgical excision (n = 35 patients; median age 25 (range 13 to 70 years)), these patients were not treated with surgical excision and were under medical treatment (200 mg bd Itraconazole or 400 mg bd ketoconazole). **Group III:** mycetoma patients who underwent surgical excision (n = 35 patients; median age 25 years (range 12 to 70 years) and medical treatment (200 mg bd Itraconazole).

**Table 1:** Distribution of gender, medical treatment, size of lesions and presence of grains among the study population.

Study groups	Controls	Mycetoma Patients		*Overall p value (95% CI)*^‡^	*p value (95% CI)*^₸^
		Without surgical excision	With surgical excision		
**Gender *n = 140***					
Female n = 49 (35%)	35 (71.4%)	8 (16.3%)	6 (12.2%)	**<0.001** (0.001 to 0.01)	0.55 (0.44 to 4.67)
Male n = 91 (65%)	35 (38.5%)	27 (29.7%)	29 (31.9%)		
**Medical treatment *n = 70***					
Itraconazole 200 mg bd n = 46 (66%)	NA*	11 (24%)	35 (76%)	**<0.001** (0.55 to 0.80)	**<0.001** (0.58 to 0.93)
Ketoconazole 400 mg bd n = 24 (34%)	NA	24 (100%)	0 (0%)		
**Size of lesions *n = 70***					
No mass n = 1 (2%)	NA	0 (0%)	1 (100%)	**<0.001** (2.04 to 16.58)	**0.037** (-0.58 to 0.13)
Less than 5 cm n = 12 (17%)	NA	7 (58.3%)	5 (41.7%)		
5–10 cm n = 33 (47%)	NA	20 (60.6%)	13 (39.4%)		
More than 10 cm n = 24 (34%)	NA	8 (33.3%)	16 (66.7%)		
**Grains *n = 70***					
None n = 52 (74%)	NA	25 (48.1%)	27 (51.9%)	**<0.001** (0.13 to 1.31)	0.28 (-0.15 to 0.27)
Present n = 18 (26%)	NA	10 (55.6%)	8 (44.4%)		

^‡^ P values are derived from comparison between the study groups using one way ANOVA test.

^₸^ P values are derived from comparison within mycetoma patients, treated with surgical excision compared to those treated without surgical excision using *Student’s t* test.

* NA not applicable

### Sample collection

One hundred μl of blood were collected on filter paper (Whatman qualitative filter paper, Grade 1, circles, diam. 42.5 mm from SIGMA-ALORICH, KSA) for cytokine’s determination. The use of filter paper dried whole blood spots (DBS) for specimen collection was preferred to facilitate collection, storage and transportation of specimens in addition to being recommended by the World Health Organization (WHO) and also used in several previous studies [7–9].

### Sera elution (extraction) from filter-paper samples

A hole puncher with a diameter of 6 mm was used for cutting out discs from the filter paper in the middle of the blood spot, where the blood was assumed to be evenly spread. The discs were put in 10 ml tubes and 500 μl of PBS containing 0.05% Tween and 0.5% BSA was added. The discs were then incubated for 2 hrs at room temperature on a shaker. Finally, after vortexing the samples for 30 seconds, the supernatants (eluted serum) were collected with a Pasteur pipette and aliquoted in new 1.5 ml cryo tubes and stored at −20°C until analysis. The extract corresponds to a serum dilution of ~1:100. This method was modified from a previous report by Mercader and colleagues [8].

### Determination of cytokines in sera samples using cytometric bead array

Measurements of cytokines were performed in sera by flow cytometry using Cytometric Bead Array (CBA) technology, as detailed by Cook and associates [10]. Human Inflammation CBA kit (BD Biosciences, San Jose, CA) was used to quantitatively measure IFN-γ, TNF-α, IL-1β, IL-2, IL-4, IL-5, IL-6, IL-10, and IL-13 levels. The sensitivity of Human Inflammation CBA was comparable to conventional ELISA [11]. Samples were analysed using a BD FACSCalibur flow cytometer (BD Biosciences, San Jose, CA), according to the manufacturer’s instructions.

### Statistical analysis

The data was managed by SPSS statistics software version 23 for Windows (IBM, SPSS statistics). The one-way analysis of variance (ANOVA) and Tukey’s test for post hoc analysis were used to compare mean levels of cytokines between various study groups. The difference in cytokine levels across groups was analysed using ANOVA test (Table 2). Linear regression models were used to predict each cytokine level (Table 3). Unstandardised coefficient (B) regression is the determination of the statistical relationship between two or more variables [12]. B analysis was adjusted for each cytokine according to gender (Female = 0 and male = 1), medical treatments (Itraconazole = 0 and Ketoconazole = 1), size of mass (>10 cm = 1), presence of grains (No = 0 and Yes = 1) and age, as independent variables.

**Table 2:** Description of median and range of cytokines (IFN-γ, TNF-α, IL-1B, IL-2, IL-4, IL-5, IL-6, IL-10 and IL-13) level/μl in different study groups.

Cytokines	Controls		Without Surgery		With Surgery		
	Mean	SD	Mean	SD	Mean	SD	P-value*
IFN-γ	4.61	1.19	21.53	7.46	27.17	10.39	<0.001
TNF-α	3.37	2.36	6.59	4.68	21.17	7.63	<0.001
IL-1 β	3.44	20.93	2.58	0.9	2.22	0.14	0.913
IL-2	0.19	0.22	9.65	2.54	17.21	5.17	<0.001
IL-4	1.01	0.35	4.99	0.77	2.42	1.53	<0.001
IL-5	0.6	1.11	3.12	1.35	1.04	0.16	<0.001
IL-6	0.98	0.41	30.61	4.49	20.52	9.63	<0.001
IL-10	3.7	1.48	21.69	2.37	16.36	6.9	<0.001
IL-13	2.46	1.02	9.73	0.36	18.37	6.32	<0.001

*p values are derived from one way ANOVA test.

**Table 3:** Linear regression analysis of circulating cytokines (IFN-γ, TNF-α, IL-1β, IL-2, IL-4, IL-5, IL-6, IL-10 and IL-13) level/μl in mycetoma patients treated with surgical excision compared to those treated without surgical excision.

Variables	IFN-γ		TNF-α		IL-1β		IL-2		IL-4		IL-5		IL-6		IL-10		IL-13	
	B*	P-value	B	P-value	B	P-value	B	P-value	B	P-value	B	P-value	B	P-value	B	P-value	B	P-value
**Unadjusted**																		
**With surgery**	5.64	0.011	14.58	<0.001	-0.36	0.022	7.55	<0.001	-2.57	<0.001	-2.08	<0.001	-10.09	<0.001	-5.33	<0.001	8.64	<0.001
**95%CI for B**	1.33 to 9.96		11.56 to 17.6		-0.67 to -0.05		5.61 to 9.50		-3.14 to -1.99		-2.54 to -1.62		-13.68 to -6.51		-7.79 to -2.87		6.51 to 10.781	
**Adjusted**																		
**With surgery**	6.62	0.013	12.69	<0.001	-0.75	<0.001	6.59	<0.001	-2.82	<0.001	-2.38	<0.001	-7.66	0.005	-3.58	0.05	9.28	<0.001
**95%CI for B**^¶^	1.42 to 11.81		9.06 to 16.32		-1.13 to -0.37		3.91 to 9.28		-3.65 to -1.99		-3.04 to -1.72		-12.88 to -2.44		-7.16 to 0.01		6.14 to 12.41	
**Male gender**	-4.1	0.077	-1.2	0.46	-0.52	0.003	-1	0.385	-0.77	0.71	-0.55	0.061	-0.57	0.805	-0.67	0.671	0.65	0.641
**age/years**	-0.13	0.104	-0.1	0.15	0.01	0.602	0.08	0.05	0.01	0.73	-0.01	0.916	0.08	0.306	-0.05	0.32	0.08	0.095
**Ketoconazole**	2.94	0.282	-1.44	0.45	-0.68	0.001	-1.51	0.285	-0.43	0.33	-0.53	0.13	4.32	0.118	2.46	0.195	0.71	0.672
**Size of lesions >10cm**	7.1	<0.001	5.23	<0.001	-0.21	0.029	0.26	0.692	-0.07	0.72	-0.12	0.44	1.82	0.153	-0.43	0.619	-1.31	0.088
**Grains**	3.55	0.086	2.27	0.12	0.13	0.378	2.96	0.006	-0.29	0.36	-0.01	0.98	-0.92	0.651	-2.31	0.104	-0.25	0.842

*B represents unstandardized coefficients

^**¶**^ **B** (95%CI) adjusted with gender, medical treatments, size of lesions and presence of grains.

### Ethical considerations

This study was approved by the Ethics Committee of Soba University Hospital, Khartoum, Sudan. Written informed consent was obtained from the participants prior to their enrolment in the study. Informed consent was also obtained from children and their guardians before participation. The work described here was performed in accordance with the Declaration of Helsinki [13].

## Results

### Distribution of gender, medical treatment, size of lesions and presence of grains in the lesions within the different study groups

A higher proportion of mycetoma patients were males (80%) compared with females (20%). Combined (both with and without surgical intervention) males and females among mycetoma patients groups were 56/70 and 14/70, respectively (*p* <0.001; Table 1).

Patients with mycetoma received various antifungal drugs, which were used in combination with or without surgical excision. Of the 70 individuals who received oral medication in this study, 46 patients (66%) received Itraconazole. Out of the patients who were treated with Itraconazole, Eleven patients (24%) were treated without surgical excision and 35 patients (76%) were surgically treated along with Itraconazole 200 mg bd. Twenty four patients (34%) received Ketoconazole 400 mg bd [*p* value <0.001 and 95% confidence interval 95%CI; (0.55 to 0.80)] (Table 1). Ketoconazole 400 mg bd was only used among patients without surgical excision and not following surgery, whereas Itraconazole 200 mg bd was the only choice postoperatively [*p* value <0.001 and 95% CI; (0.58 to 0.93)].

The proportion of lesions that were more than 10 cm in diameter were significantly higher in the surgically treated group compared to the non-surgically treated patients [*p* value = 0.037 and 95% CI; (-0.58 to 0.13)] (Table 1).

### Cytokine levels in the different study groups

Patients with mycetoma infection had significantly higher cytokine levels including IFN-γ, TNF-α, IL-2, IL-4, IL-5, IL-6, IL-10 and IL-13, compared to the control group (overall *p* value for each cytokine <0.001) (Table 2). In contrast; no significant difference was observed in the levels of IL-1β between the study groups (overall *p* value = 0.913)(Table 2).

Linear regression analysis showed significantly higher levels of Th-1 cytokines (IFN-γ, TNF-α, IL-1β and IL-2) among mycetoma patients treated with surgical excision than in those treated without surgical intervention. Unadjusted B (95% CI) for: IFN-γ = [5.64; 95% CI (1.33 to 9.96), *p* value = 0.011]. For TNF-α = [14.58; 95% CI (11.56 to 17.60), *p* value <0.001]. For IL-1β = [-0.36; 95% CI (-0.67 to -0.05), *p* value = 0.022]. For IL-2 = [7.55; 95% CI (5.61 to 9.50), *p* value <0.001] (Table 3).

When B was adjusted for gender, medical treatment, size of lesions and the presence of grains; similar statistical analysis indicated significantly higher levels of Th-1 cytokines (IFN-γ, TNF-α, IL-1β and IL-2) among mycetoma patients treated with surgical excision than in those treated without surgical excision. Adjusted B (95% CI) for: IFN-γ = [6.62; 95% CI (1.42 to 11.81), *p* value = 0.017]. For TNF-α = [12.69; 95% CI (9.94 to 16.32), *p* value <0.001]. For IL-1β = [-0.75; 95% CI (-1.13 to -0.37), *p* value <0.001]. For IL-2 = [6.59; 95% CI (3.91 to 9.28), *p* value <0.001] (Table 3).

In contrast, a similar linear regression analysis model for Th-2 cytokines showed significantly lower levels of Th-2 cytokines (IL-4, IL-5, IL-6 and IL-10) among mycetoma patients treated with surgical excision, compared to those treated without surgical excision. Unadjusted B (95% CI) for: IL-4 = [-2.57; 95% CI (-3.14 to -2.0), *p* value <0.001]. For IL-5 = [-2.08; 95% CI (-2.54 to -1.62), *p* value <0.001]. For IL-6 = [-10.09; 95% CI (-13.68 to -6.51), *p* value <0.001]. For IL-10 = [-5.33; 95% CI (-7.79 to -2.87), *p* value <0.001] (Table 3).

When B was adjusted for gender, medical treatment, size of lesions and presence of grains, a similar statistical analysis model showed significantly lower levels of Th-2 cytokines (IL-4, IL-5, IL-6 and IL-10) among mycetoma patients treated with surgical excision compared to those treated without surgical excision (Table 3). Adjusted B (95% CI) for: IL-4 = [-2.82; 95% CI (-3.65to -1.99), *p* value <0.001]. For IL-5 = [-2.38; 95% CI (-3.04 to -1.72), *p* value <0.001]. For IL-6 = [-7.66; 95% CI (-12.88 to -2.44), *p* value = 0.005]. For IL-10 = [-3.58; 95% CI (-7.16 to -0.01), *p* value = 0.05] (Table 3).

## Discussion

It is known that fungi release antigens (Ag) on the skin surface, and the antigens that penetrate the skin are subsequently captured by an antigen-presenting cell (APC) such as dendritic cells (DCs) [14]. Fungal antigens can also play an important role in the DCs maturation. Furthermore, production of inflammatory cytokines such as IFN-γ and TNF-α by other innate cells such as natural killer cells (NK) further enhance the activation of microbiocidal functions of phagocytic cells as well as maturation of DCs [15].

In the present study, Th-1 cytokines (IFN-γ, TNF-α, and IL-2) were found to be significantly higher in mycetoma patients than in controls. Besides, the levels of Th-1 (IFN-γ, TNF-α, IL-1β and IL-2) were significantly higher in mycetoma patients treated with surgical excision compared to those who were only medically treated. These findings go a long way to explain the earlier findings of van de Sande and associates [16], that neutrophils are attracted to the site of infection by mycetoma antigen, secrete TNF-α and IFN-γ cytokines in the presence of IL-17 [17]. Interestingly, in a previous study, Cassatella and colleagues suggested that neutrophils are multipurpose cells which play many roles, not only in inflammatory progressions but also in immune and antitumor processes [18]. The same group had also added that, IFN-γ activated neutrophils release biologically active TNF-α related apoptosis-inducing ligand (TRAIL/APO2 ligand), a molecule that exerts selective apoptotic activities towards tumours [18]. Additionally, Elagab and associates, showed that, the peripheral blood mononuclear cells (PBMC) of mycetoma patients react differently *to M*. *mycetomatis* antigens than healthy controls [19]. In general, when PBMCs produce IFN-γ upon stimulation with the antigen, no production of IL-10 was detected [19]. There is also no significant differences between the cytokines TNF-α and TGF-β levels in patients and controls [19]. The discrepancy between Elagab’s findings [19] and our findings may be explained by the differences in the study design.

IL-1 is an essential host defence cytokine against a broad range of pathogens, ranging from bacteria to parasites and fungi [20]. IL-1β is primarily produced by innate immune cells such as monocytes, macrophages and dendritic cells upon activation, and is also an important cytokine for the control of fungal infection [21]. It is also an important proinflammatory mediator whose production is controlled by multiprotein complexes called inflammasomes [22, 23]. Although IL-1β plays an active role in containing infection caused by different fungi, its role in controlling fungal infections remains unclear [24]. The results of the current study has shown that higher levels of IL-1β cytokine are strongly associated with mycetoma patients treated with surgical excision, compared to those treated without surgical intervention. It is of interest to note that, IL-1β can play a crucial role in the activation of complement protein-3 (CR3), dectin-1 as well as caspase-8 in coordinating cell death and inflammasome responses to β-glucans [25]. Our findings led us to suggest that the observed higher levels of IL-1β cytokine play an important role in reducing the risk of *M*. *mycetomatis* infection. However, more studies are needed to confirm farther this observation.

As mentioned earlier cytokine IL-2 exerts critical functions during immune homeostasis *via* its effects on T_reg_ cells, and by optimising the effector lymphocyte responses of both T-cells and B-cells. In addition, IL-2 receptors (IL-2R) were shown to be present on human neutrophils, and that IL-2-neutrophil interactions are believed to be important in both tumour rejection and increased susceptibility to bacterial infections [26, 27]. It is relevant to add that a previous study on mycetoma patients from an endemic area [16], demonstrated that neutrophils are attracted to the site of infection by the mycetoma antigen. In the current study IL-2 levels were significantly higher in mycetoma patients compared to controls. In addition, IL-2 cytokine levels were elevated significantly in mycetoma patients treated with surgical excision, compared to those treated without surgical intervention. We take this finding to indicate that, IL-2 cytokine plays a major role in the pathogenesis of mycetoma infection. This novel finding on an association of IL-2 and neutrophils should pave the way to new avenues of research on IL-2-neutrophil interactions to better understand the response of patients to mycetoma infection.

The cytokines IL-4, IL-5, IL-13 and GM-CSF are produced by T-helper-2 cells at the site of inflammation but also they have important functions in haematopoiesis. These cytokines, individually or collectively along with chemokines such as CCL11, play a major role in coordinating the maturation and mobilisation of leukocytes (Monocytes/Macrophages and Neutrophils) and mast cell progenitors, ensuring the continued supply of leukocytes to the site of the inflammation [28, 29].

In present study, the *in vivo* effect of *M*. *mycetomatis* infection on the production of Th-2 cytokines (IL-4, IL-5, IL-6 and IL-10) was clearly reflected by the, significantly higher levels of Th-2 cytokines in mycetoma patients compared to controls. Moreover, lower levels of Th-2 cytokines (IL-4, IL-5, IL-6 and IL-10) were significantly associated with mycetoma patients treated with surgical excision, compared to those treated without surgical intervention. This finding is in line with the earlier hypothesis that Th-2 cytokines play an important role in the activation of the humoral immune response [28, 29].

It is well stablished that the type of cell-mediated immunity (CMI) is critical in determining resistance or susceptibility to fungal infection. In general, Th1-type CMI is required for the clearance of fungal infections, while Th2 immunity usually enhances the susceptibility to infection and allergic responses [30]. Additionally, Th-1 cells are concerned mainly with production of cytokines such as IFN-γ, and promote CMI and phagocyte activation, while in contrast, Th-2 cells predominantly produce cytokines such as IL-4 and IL-5 and tend to promote antibody production [30–32]. Besides, IL-4 and IL-5 cytokines can play an important role in the activation of B-cells to differentiate to plasma cells that secrete IgM antibody and also generate memory B cells [33].

A previous similar study found elevated levels of IgM antibody in mycetoma patients [5]. Besides, another study on immune responses against mycetoma Sudanese patients, demonstrated the presence of immunoglobulins G, M and complement on the surface of the grains and on the filaments inside the grains of mycetoma lesions [34]. Also, both neutrophils and macrophages were recruited into the lesion by complement and were involved in the fragmentation of the grains. The cytokines profile in the lesion and regional lymph nodes was of a dominant Th-2 pattern (IL-10 and IL-4) [34], and these elevated levels of Th-2 cytokines in mycetoma patients may trigger the increased production of IgG, IgM and complement. The significance of this phenomenon needs further investigations.

We noted with great interest higher levels ofTh-1 cytokines (IFN-γ, TNF-α, IL-1β and IL-2) in mycetoma patients treated with surgical excision than in those patients treated without surgical intervention. However, in contrast the Th-2 cytokines (IL-4, IL-5, IL-6 and IL-10) were significantly lower in patients treated with surgical excision compared to those treated without surgical intervention.

These results suggest that, the defence against the fungus *M*. *mycetomatis* is based on the adaptive effector phase and the duration of the infection as well as the size of the mycetoma mass and presence of grains. The effects of CMI can also play a critical role in reducing the risk of localised infection in mycetoma patients treated with surgical excision compared to those treated without surgical intervention. The essential role of the CMI response is to destroy the fungi and produce an immuno-protective status against infection. At this moment the exact explanation of this finding is not clear and requires further investigation in mycetoma patients.

## Supporting Information

S1 Checklist STROBE checklist. (DOC)
